# Phase II trial of daily S‐1 combined with weekly irinotecan in previously treated patients with advanced or recurrent squamous cell lung cancer: North Japan lung cancer group 1101

**DOI:** 10.1111/1759-7714.15076

**Published:** 2023-08-17

**Authors:** Yosuke Kawashima, Osamu Ishimoto, Eisaku Miyauchi, Tomohiro Sakakibara, Toshiyuki Harada, Kazuhiro Usui, Akira Inoue, Shunichi Sugawara

**Affiliations:** ^1^ Department of Pulmonary Medicine Sendai Kousei Hospital Sendai Japan; ^2^ Okino Medical Clinic Sendai Japan; ^3^ Department of Respiratory Medicine Tohoku University Graduate School of Medicine Sendai Japan; ^4^ Department of Respiratory Medicine Tohoku Rosai Hospital Sendai Japan; ^5^ Department of Respiratory Medicine JCHO Hokkaido Hospital Sapporo Japan; ^6^ Division of Respirology NTT Medical Center Tokyo Tokyo Japan; ^7^ Department of Palliative Medicine Tohoku University Graduate School of Medicine Sendai Japan

**Keywords:** carcinoma, clinical trial, irinotecan, non‐small cell lung, phase II

## Abstract

**Background:**

This phase II trial was designed to evaluate the efficacy and safety of S‐1 combined with weekly irinotecan as a second‐ or third‐line treatment for patients with advanced or recurrent squamous cell lung cancer.

**Methods:**

Patients with a body surface area <1.25, 1.25–1.50, and >1.50 m^2^ received oral S‐1 on days 1–14 at 80, 100, and 120 mg/day, respectively, and irinotecan on days 1 and 8 at 70 mg/m^2^ every 3 weeks. The primary endpoint was the overall response rate, and the secondary endpoints were progression‐free survival, overall survival, and the incidence and severity of adverse effects.

**Results:**

Between September 2011 and December 2014, 30 patients were enrolled in this study. The overall response rate was 6.7% (95% confidence interval [CI]: 0.8%–22.1%), and the disease control rate was 73.3%. The median progression‐free survival was 3.0 months (95% CI: 2.5–3.4 months), and the median overall survival was 10.5 months (95% CI: 5.6–13.7 months). Grade 3/4 treatment‐related adverse events were reported in ≥10% of the patients, including leukopenia (21%), neutropenia (21%), anemia (17%), anorexia (10%), and hypokalemia (10%).

**Conclusions:**

Although the treatment‐related adverse events were manageable, the combination of weekly irinotecan and S‐1 did not have the expected effect.

## Graphical abstract

- The combination of weekly irinotecan and S‐1 was not superior to single‐agent therapy in terms of ORR, although the toxicity profile was as expected.
- This combination did not yield the expected effects and may not be a candidate regimen for future phase III trials.

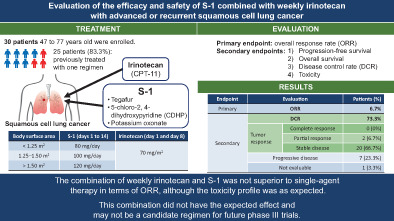

## INTRODUCTION

Lung cancer is one of the leading causes of cancer‐related deaths both in Japan and worldwide.^1^ Non‐small cell lung cancer (NSCLC) accounts for 85% of all lung cancers, and squamous cell carcinoma is the second most common subtype after adenocarcinoma. Squamous cell carcinoma accounts for 40% and 15% of male and female lung cancer cases, respectively.

For patients with NSCLC previously treated with platinum‐doublet chemotherapy, single‐agent chemotherapy, such as pemetrexed and docetaxel, is recommended. However, in terms of efficacy, pemetrexed is not recommended for patients with squamous cell carcinoma.^2^ Furthermore, the efficacy of docetaxel is unsatisfactory, and optimization of treatment strategies for patients with squamous cell carcinoma is required.^3^

S‐1 is a novel oral fluoropyrimidine agent consisting of tegafur, 5‐chloro‐2, 4‐dihydroxypyridine (CDHP), and potassium oxonate at a molar ratio of 1:0.4:1. Tegafur is a prodrug of 5‐fluorouracil (5‐FU), whereas CDHP inhibits dihydropyrimidine dehydrogenase, the enzyme responsible for degrading 5‐FU. Compared with tegafur alone, tegafur plus CDHP increases 5‐FU concentration in the serum and tumor tissue. Potassium oxonate is expected to alleviate the gastrointestinal toxicity of tegafur. S‐1 is commonly used for the treatment of gastrointestinal, head and neck, NSCLC, breast, pancreatic, and biliary tract cancers. In a phase II trial^4^ of S‐1 monotherapy in patients with advanced NSCLC without prior chemotherapy, the overall response rate (ORR) was 22.0% (95% confidence interval [CI]: 12.3%–34.7%) without any irreversible, severe, or unexpected toxicities. Another phase II trial^5^ of S‐1 monotherapy as a second‐line treatment for NSCLC reported an ORR of 12.5% (95% CI: 3.1%–21.9%), with a median overall survival (OS) of 8.2 months and acceptable toxicity. An additional phase II trial^6^ of cisplatin combined with S‐1 in patients with advanced NSCLC without prior chemotherapy reported an ORR of 47% (95% CI: 34%–61%), with a median OS of 11 months and acceptable toxicity. A randomized phase III trial^7^ of S‐1 combined with carboplatin versus carboplatin combined with paclitaxel showed the noninferiority of S‐1 and carboplatin in terms of OS (15.2 vs. 13.3 months, respectively; hazard ratio [HR]: 0.928 [99.2% CI: 0.671–1.283]). These findings indicate that S‐1 has good antitumor activity against NSCLC, irrespective of whether it is administered as monotherapy or combination therapy, and is also a suitable candidate as a nonplatinum chemotherapeutic agent for patients with NSCLC.

Irinotecan (CPT‐11), an inhibitor of DNA topoisomerase I, is used in the treatment of NSCLC and has a different mechanism of antitumor activity than that of S‐1. Two randomized phase III trials^8^, ^9^ of CPT‐11 combined with cisplatin for advanced NSCLC showed comparable survival to cisplatin plus vindesine and concluded that a CPT‐11‐containing regimen is one of the most active and well‐tolerated regimens for the treatment of advanced NSCLC. A randomized phase II trial^10^ comparing CPT‐11 combined with docetaxel and cisplatin combined with docetaxel for advanced NSCLC showed no significant differences in OS between the groups. Thus, CPT‐11 combined with docetaxel may be a reasonable treatment option for patients with NSCLC who cannot tolerate cisplatin.

It has been suggested that thymidylate synthase (TS) expression levels in squamous cell carcinoma are higher than those in adenocarcinoma,^11^ and high TS expression levels contribute to the attenuation of the antitumor effect.^12^ In fact, pemetrexed, which targets TS and exerts antitumor effects, is not recommended for the treatment of squamous cell carcinoma because of its attenuation of the antitumor effect.^2^ S‐1 also targets TS; however, there is no difference in efficacy between histological types when S‐1 is combined with platinum.^7^, ^13^ For this reason, it has been suggested that 5‐FU has another mechanism involving RNA dysfunction by converting orotate phosphoribosyl transferase (OPRT) to fluorouridine monophosphate (FUMP), and high OPRT levels contribute to the enhancement of antitumor effects in metastatic colorectal cancer.^14^ According to an investigation of OPRT levels in lung cancer,^15^ OPRT levels in squamous cell carcinoma were significantly higher than those in adenocarcinoma. Therefore, S‐1 is considered to inhibit RNA dysfunction, particularly in squamous cell carcinoma. Furthermore, TS and topoisomerase I expression are positively correlated,^16^ and TS expression was reduced by CPT‐11 in a human colorectal cancer cell line in a preclinical model.^17^ In patients with gastric cancer treated with a regimen containing S‐1, high TS expression levels did not predict an antitumor effect when combined with CPT‐11, and tumors with high TS levels might respond to additional CPT‐11.^18^ Based on these data, several studies^19^, ^20^ using a combination of S‐1 and CPT‐11 demonstrated high efficacy and safety against advanced gastric and colorectal cancers. These findings led us to investigate the potential of CPT‐11 combined with S‐1 in patients with advanced NSCLC, especially squamous cell carcinoma. We have previously reported the results of a phase I trial^21^ of daily S‐1 combined with weekly CPT‐11 in patients with advanced NSCLC, and the recommended dose of CPT‐11 was 70 mg/m^2^. Here, we report the results of a phase II trial of daily S‐1 (80 mg/m^2^, days 1–14) combined with weekly CPT‐11 (70 mg/m^2^, days 1 and 8) in patients previously treated for advanced squamous cell lung cancer.

## METHODS

### Ethical considerations

All procedures involving human participants performed in this study were in accordance with the ethical standards of the institutional and/or national research committee and the 1964 Declaration of Helsinki and its later amendments or comparable ethical standards. The study design was approved by the Institutional Review Board of each participating institution. Written informed consent was obtained from all participants prior to enrollment. The study was registered in the University Hospital Medical Information Network Clinical Trials Registry (UMIN000006065).

### Patient eligibility

The main eligibility criteria were: (1) histologically or cytologically confirmed squamous cell lung cancer with a predominant squamous component, or adenosquamous carcinoma with a predominant squamous component; (2) unresectable stage III disease without an indication for curative irradiation, stage IV disease, or postoperative recurrence; (3) age >20 years; (4) measurable lesions according to the Response Evaluation Criteria in Solid Tumors (RECIST) (version 1.1);^22^ (5) previously treated with fewer than two regimens (including at least one platinum‐based regimen; epidermal growth factor receptor‐tyrosine kinase inhibitors were counted as one regimen and postoperative chemotherapy was excluded); (6) radiologically confirmed progressive disease after previous treatment (4 weeks or more after previous treatment, 4 weeks or more after chemoradiotherapy for locally advanced squamous cell lung cancer, and 2 weeks or more after palliative local radiotherapy, except for the primary lesion); (7) Eastern Cooperative Oncology Group performance status (PS) of 0/1; (8) adequate organ function (defined as absolute neutrophil count ≥1500/mm^3^, platelet count ≥100 000/mm^3^, hemoglobin ≥9.0 g/dL, aspartate aminotransferase ≤2.5 × the institutional upper limit of the normal value, alanine aminotransferase ≤2.5 × the institutional upper limit of the normal value, total serum bilirubin ≤1.5 × the institutional upper limit of the normal value, creatinine ≤1.2 mg/dL, creatinine clearance ≥60 mL/min, PaO_2_ ≥ 60 Torr, or SpO_2_ ≥ 94%); and (9) written informed consent.

The main exclusion criteria were: (1) evidence of interstitial pneumonia on chest radiography; (2) a history of drug hypersensitivity; (3) active double cancer; (4) pleural, peritoneal, and pericardial effusion requiring drainage; (5) serious complications (symptomatic cardiovascular disease, uncontrolled hypertension/diabetes mellitus, and active infection); (6) symptomatic brain metastases; (7) previously treated with S‐1, uracil‐tegafur, and CPT‐11; (8) watery diarrhea; (9) intestinal paralysis or intestinal obstruction; (10) treated with flucytosine; (11) treated with atazanavir sulfate; and (12) women who were pregnant, intending to become pregnant, or breast‐feeding.

### Treatment schedule

Every 21‐day cycle, intravenous CPT‐11 (70 mg/m^2^) was administered on days 1 and 8, and oral S‐1 was administered twice daily after meals from days 1 to 14. The dose of S‐1 was modified according to body surface area (BSA) as follows: 80 mg/day for patients with BSA <1.25 m^2^, 100 mg/day for those with BSA 1.25–1.50 m^2^, and 120 mg/day for those with BSA >1.50 m^2^. The criteria for initiating treatment were provided, and cycle delays of up to 3 weeks were permitted. The criteria for administering CPT‐11 on day 8 were provided, and the administration of CPT‐11 was omitted if the patient did not meet these criteria. The criteria for S‐1 cessation and resumption were also provided. The dose reduction criteria were as follows: for the first dose reduction, S‐1 was reduced from 120 to 100 mg/day, 100 to 80 mg/day, or 80 to 50 mg/day; for the second dose reduction, CPT‐11 was reduced from 70 to 60 mg/m^2^. Patients who required dose reduction received the reduced dose for the remainder of the study period. If a patient who required a second dose reduction became eligible for further dose reduction, they were withdrawn from the study.

### Assessment of endpoints

The primary endpoint was investigator‐assessed ORR, defined as the proportion of confirmed complete or partial responses. Tumor response was assessed using spiral computed tomography and evaluated using RECIST (version 1.1)^22^ every 4 weeks from screening until progressive disease occurred. After the discontinuation of treatment, tumor response was confirmed as complete response, partial response, or stable disease, and computed tomography was performed every 8 weeks. The secondary endpoints were progression‐free survival (PFS), OS, disease control rate (DCR), and toxicity. PFS was defined as the time from randomization to disease progression according to the RECIST (version 1.1)^22^ or death due to any cause, whichever occurred first. OS was defined as the time from randomization to death due to any cause. DCR was defined as the proportion of complete response, partial response, or stable disease according to the RECIST (version 1.1).^22^ Toxicities were assessed according to the Common Terminology Criteria for Adverse Events (version 4.0). The investigators determined whether the trial regimen caused any toxicities.

### Statistical analysis

Several phase III trials of docetaxel and erlotinib in patients previously treated with platinum have shown an ORR ranging from 5.5% to 12.8%. Combination therapy containing CPT‐11 administered to patients previously treated with platinum resulted in an ORR ranging from 10% to 20%. Based on these findings, we assumed that an ORR of 20% in eligible patients indicated potential usefulness, and an ORR of 5% was the lower limit of interest. Accordingly, the estimated accrual number was 28 patients, using the Southwest Oncology Group one‐arm binomial method (one‐sided alpha = 0.05; beta = 0.20). After adjusting for dropouts, the actual accrual number was 30 patients. PFS and OS were estimated until June 30, 2015 using the Kaplan–Meier method and compared using the log‐rank test. All statistical analyses were conducted using the EZR software (Saitama Medical Center, Jichi Medical University, Saitama, Japan).

## RESULTS

### Patient characteristics

Thirty patients were enrolled between September 2011 and December 2014. The baseline characteristics of the patients are summarized in Table 1. The median age was 65 years (range, 47–77 years), and 23 patients (76.7%) were men. Twenty‐five patients (83.3%) were previously treated with one regimen.

**TABLE 1:** Patient characteristics.

Characteristic	Patients (*n* = 30)
Age (years), median (range)	65 (47–77)
Sex, n (%)	
Male	23 (76.7)
Female	7 (23.3)
Performance status (ECOG), n (%)	
0	16 (53.3)
1	14 (46.7)
Clinical stage, n (%)	
III	11 (36.7)
IV	16 (53.3)
Postoperative recurrent disease	3 (10.0)
Number of previous regimens, n (%)	
1	25 (83.3)
2	5 (16.7)

Abbreviation: ECOG, Eastern Cooperative Oncology Group.

### Treatment delivery

At the time of data cutoff (June 30, 2015) for the final analysis, all patients had completed treatment. The median number of treatment cycles was three (range: 1–18). Three patients (10.0%) required a dose reduction of CPT‐11, and 15 patients (50.0%) required a delay in treatment cycles. The administration of CPT‐11 on day 8 was omitted in three patients (10.0%). Six patients (20.0%) required a dose reduction of S‐1, while seven patients (23.3%) required cessation of S‐1 in the treatment cycles (Table 2). The median relative dose intensities were 83.3% (CPT‐11) and 84.9% (S‐1), respectively.

**TABLE 2:** Treatment delivery.

Variable	S‐1	CPT‐11
Dose reduction, n (%)	6 (20.0)	3 (10.0)
Course delay, n (%)	15 (50.0)	15 (50.0)
Cessation, n (%)	7 (23.3)	–
Omitted CPT‐11 on day 8, n (%)	–	3 (10.0)
Median relative dose intensity (%)	84.9	83.3

*Note*: The median number of treatment cycles was three (range: 1–18).

### Efficacy

**FIGURE 1:**
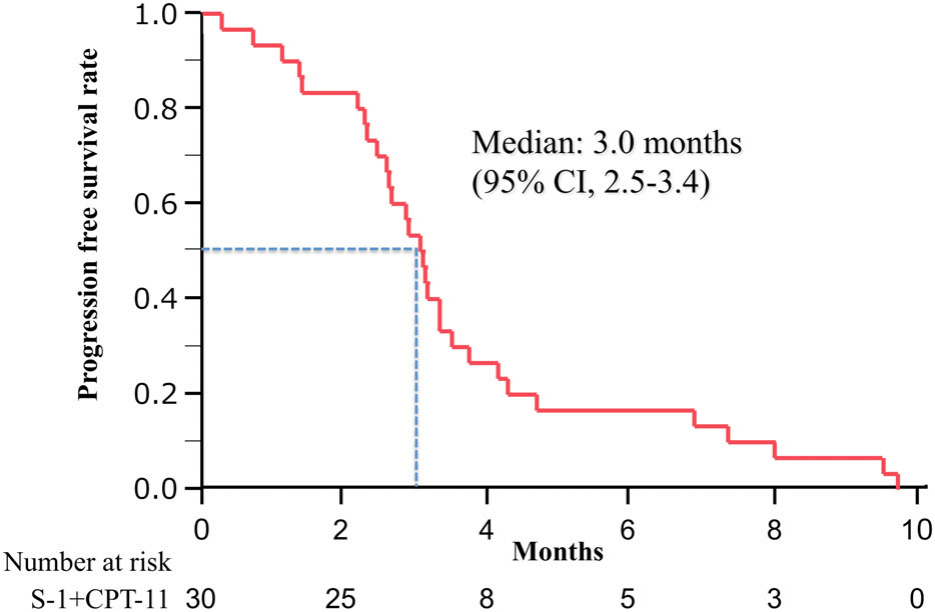
Kaplan–Meier curve of progression‐free survival. Ticks indicate the patients for whom data were censored on June 30, 2015. CI, confidence interval; CPT‐11, irinotecan.

**FIGURE 2:**
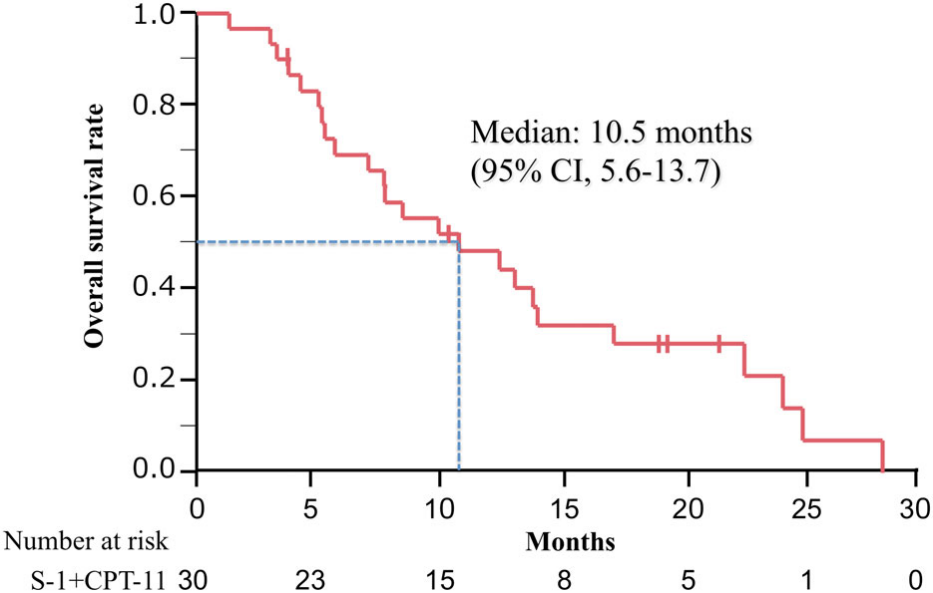
Kaplan–Meier curve of overall survival. Ticks indicate the patients for whom data were censored on June 30, 2015. CI, confidence interval; CPT‐11, irinotecan.

**FIGURE 3:**
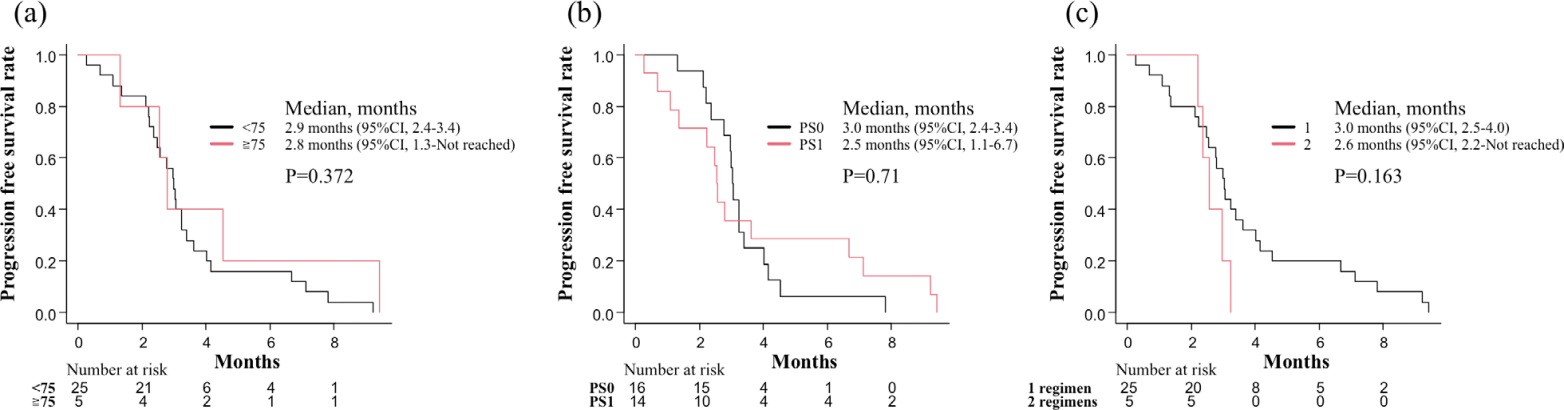
Kaplan–Meier curve of progression‐free survival classified by age (a), PS (b), and previous regimen number (c). Ticks indicate the patients for whom data were censored on June 30, 2015. CI, confidence interval; PS, performance status.

**FIGURE 4:**
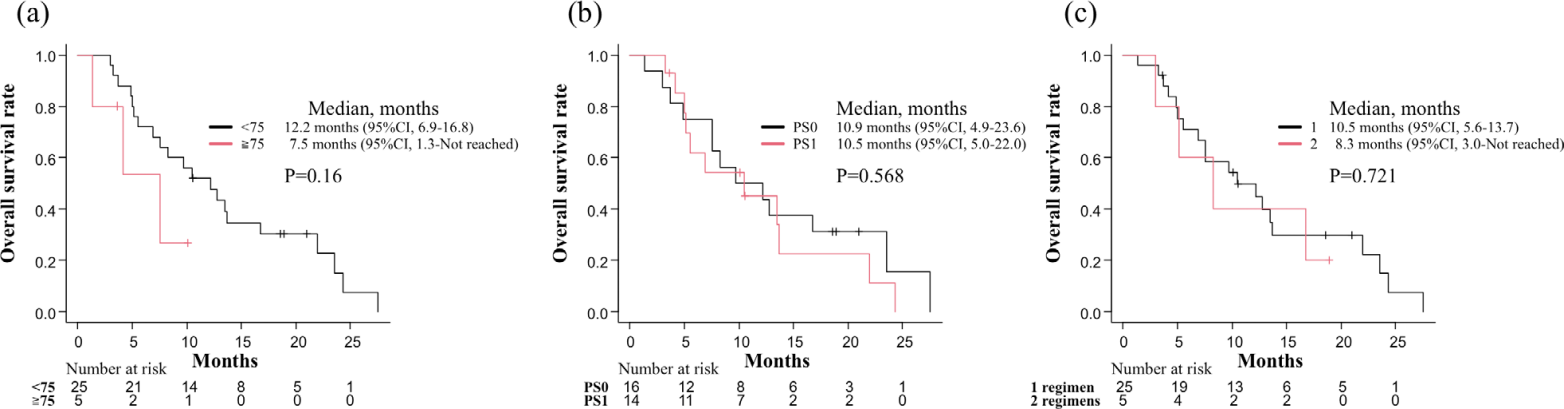
Kaplan–Meier curve of overall survival classified by age (a), PS (b), and previous regimen number (c). Ticks indicate the patients for whom data were censored on June 30, 2015. CI, confidence interval; PS, performance status.

The tumor response was evaluated in all patients enrolled in this study. The ORR was 6.7% (95% CI: 0.8%–22.1%), whereas the DCR was 73.3% (95% CI: 54.1–87.7%). The tumor responses are summarized in Table 3. The median PFS was 3.0 months (95% CI: 2.5–3.4 months) (Figure 1), and the median OS was 10.5 months (95% CI: 5.6–13.7 months) (Figure 2). Subgroup analyses of PFS (Figure 3) and OS (Figure 4) classified by age (a), PS (b), and the number of previous regimens (c) showed no significant differences with respect to these factors.

**TABLE 3:** Response to treatment.

Response	Patients (n)	(%)
Complete response	0	0
Partial response	2	6.7
Stable disease	20	66.7
Progressive disease	7	23.3
Not evaluable	1	3.3
Overall response rate (%)	–	6.7 (95% CI: 0.8–22.1)
Disease control rate (%)	–	73.3 (95% CI: 54.1–87.7)

Abbreviation: CI, confidence interval.

### Safety

One patient was excluded because of lack of data, and 29 patients were included in the safety analysis. The adverse events are listed in Table 4. Grade 3/4 hematological toxicities included leukopenia (21%), neutropenia (21%), and anemia (17%). Grade 3/4 nonhematological toxicities included anorexia (10%), nausea (3%), diarrhea (7%), oral mucositis (3%), peripheral neuropathy (3%), febrile neutropenia (3%), hypoalbuminemia (3%), hyponatremia (7%), and hypokalemia (10%). There were no treatment‐related deaths.

**TABLE 4:** Hematological and nonhematological toxicities.

Toxicity	All n (%)	Grade 3 n (%)	Grade 4 n (%)	Grade 3/4 (%)
Hematological				
Leucopenia	10 (34.5)	5 (17.2)	1 (3.4)	20.7
Neutropenia	12 (41.4)	1 (3.4)	5 (17.2)	20.7
Anemia	26 (89.7)	5 (17.2)	0 (0.0)	17.2
Thrombocytopenia	9 (31.0)	0 (0.0)	0 (0.0)	0.0
Nonhematological				
Fatigue	3 (10.3)	0 (0.0)	0 (0.0)	0.0
Anorexia	15 (51.7)	3 (10.3)	0 (0.0)	10.3
Nausea	8 (27.6)	1 (3.4)	0 (0.0)	3.4
Diarrhea	11 (37.9)	2 (6.9)	0 (0.0)	6.9
Oral mucositis	2 (6.9)	1 (3.4)	0 (0.0)	3.4
Peripheral neuropathy	2 (6.9)	1 (3.4)	0 (0.0)	3.4
Febrile neutropenia	1 (3.4)	1 (3.4)	0 (0.0)	3.4
Hypoalbuminemia	6 (20.7)	1 (3.4)	0 (0.0)	3.4
Elevated creatinine	5 (17.2)	0 (0.0)	0 (0.0)	0.0
Hyponatremia	4 (13.8)	2 (6.9)	0 (0.0)	6.9
Hypokalemia	7 (24.1)	2 (6.9)	1 (3.4)	10.3

## DISCUSSION

In this study, we evaluated the efficacy and safety of CPT‐11 and S‐1 combination in patients with previously treated advanced or recurrent squamous cell lung cancer. We obtained an ORR of 6.7%, whereas the median PFS and OS were 3.0 months and 10.5 months, respectively. To the best of our knowledge, only two studies have evaluated the efficacy and safety of CPT‐11 and S‐1 combination in patients with previously treated advanced or recurrent NSCLC. Goya et al.^23^ reported that the ORR was 15.8% (90% CI: 6.1%–25.5%), whereas the median PFS and OS were 4.5 months (95% CI: 3.5–5.0 months) and 15.0 months (95% CI: 9.5–20.6 months), respectively. Ikeumura et al.^24^ reported that the ORR was 6.5% (95% CI: −2.6%–15.5%), whereas the median PFS and OS were 2.8 months (95% CI: 2.3–3.4 months) and 12.6 months (95% CI: 8.9–19.9 months), respectively. In both studies, the dominant histological type was adenocarcinoma, and a limited number of patients with squamous cell lung cancer were included (15.8% and 22.6%, respectively).^23^, ^24^ Therefore, in this study, we evaluated the efficacy and safety of CPT‐11 and S‐1 combination in patients with squamous cell lung cancer.

Although we had planned that CPT‐11 would be administered at a dose of 70 mg/m^2^ on days 1 and 8 every 3 weeks (i.e., 46.7 mg/m^2^/week) according to the results of a phase I trial^21^ of daily S‐1 combined with weekly CPT‐11 in patients with advanced NSCLC, the dominant histological type was adenocarcinoma, and only one patient (7.7%) with squamous cell lung cancer was included in the trial. In this study, the median relative dose intensity and the actual dose intensity of CPT‐11 was 83.3% and 38.9 mg/m^2^/week, respectively. The histological type and discrepancy in the dose intensity of CPT‐11 may have contributed to the lower ORR than expected.

The toxicity profiles of adverse events were as expected. For instance, 6/29 patients (20.7%) experienced grade 3/4 leukopenia or neutropenia, which was consistent with the results of previous studies on second‐line treatment. In these studies, the incidence of grade 3/4 neutropenia in patients treated with combined CPT‐11 and S‐1 was 9.7%–17.9%. With respect to nonhematological toxicities, grade 3/4 diarrhea occurred in 2/29 patients (6.9%), which was within the acceptable range. Of note, 5/29 patients (17.2%) experienced grade 3/4 anemia. Although no patients developed grade 4 anemia, we should keep in mind that anemia can sometimes be life‐threatening.

This study had several limitations. First, immunotherapy was introduced into clinical practice after this trial, and no patients were initially treated with immunotherapy. Immune checkpoint inhibitor (ICI) monotherapy, ICIs plus chemotherapy, and ICI combination therapy are currently the standard treatments for advanced NSCLC. Therefore, our study does not reflect recent advances in immunotherapy. Interestingly, the combination of S‐1 and/or CPT‐11 with immunotherapy may be a candidate regimen for other cancer because the synergy effects of these drugs have been reported.^25^, ^26^, ^27^ In fact, pembrolizumab combined with S‐1 based regimen has shown favorable efficacy and manageable safety as first‐line treatment in patients with gastric cancer.^28^ It is just a guess that CPT‐11 and S‐1 combination may have different effects after immunotherapy. Second, we planned this study based on the hypothesis that OPRT and TS expression in squamous cell carcinoma differs from that in adenocarcinoma. However, we did not evaluate the actual expression of OPRT or TS. Third, although this combination regimen is applicable in Japanese clinical practice, it may not be considered in countries outside Japan.

In conclusion, CPT‐11 and S‐1 combination was not superior to single‐agent therapy in terms of ORR, although the toxicity profile was as expected. This combination did not yield the expected effect and may not be a candidate regimen for future phase III trials.

## AUTHOR CONTRIBUTIONS

Shunichi Sugawara was the principal investigator. Shunichi Sugawara, and Yosuke Kawashima contributed to the study design, data analysis, and data interpretation.

Yosuke Kawashima, Osamu Ishimoto, Eisaku Miyauchi, Tomohiro Sakakibara, Toshiyuki Harada, Kazuhiro Usui, Akira Inoue, and Shunichi Sugawara contributed to patient recruitment and data collection. Yosuke Kawashima, and Shunichi Sugawara prepared the initial draft of the report with input from other authors. All authors approved the final version of the report.

## CONFLICT OF INTEREST STATEMENT

Eisaku Miyauchi received personal fees from Taiho Pharmaceutical Co., Ltd. outside of the submitted work. Akira Inoue received personal fees from Daiichi Sankyo Co., Ltd. outside of the submitted work. Shunichi Sugawara received personal fees from Taiho Pharmaceutical Co., Ltd., Chugai Pharma, AstraZeneca, MSD, Bristol‐Myers Squibb, Ono Pharmaceutical Co., Ltd., Nippon Boehringer Ingelheim Co., Ltd., Pfizer, Eli Lilly & Co., Novartis, Kyowa Kirin Co., Ltd., and Yakult Honsha Co., Ltd. outside of the submitted work. None of the remaining authors declare any conflicts of interest.

## Data Availability

All data generated and/or analyzed during this study are included in this published article.
